# Cyclin‐dependent kinase 1 shows to be a potential genetic target for chemical cystitis

**DOI:** 10.1002/iid3.454

**Published:** 2021-06-03

**Authors:** Kun Wang, Huaping Du, Qianfeng Zhuang, Hao Lu, Renfang Xu, Dong Xue

**Affiliations:** ^1^ Department of Surgical Urology The Third Affiliated Hospital of Soochow University Changzhou Jiangsu China; ^2^ Department of Neurology Suzhou Ninth People's Hospital Suzhou Jiangsu China

**Keywords:** bioinformatics, Cdk1, chemical cystitis, P53, pathogenic gene

## Abstract

**Background:**

In the present study, we aimed to explore whether common genetic targets or signaling pathways existed in chemical cystitis.

**Methods:**

Gene Expression Omnibus (GEO) database was used to search the related gene expression profiles. The differentially expressed genes (DEGs) were identified by using GEO2R. The DAVID 6.8 Beta and R software were used to perform Kyoto Encyclopedia of Genes and Genomes pathway analysis and Gene Ontology function analysis of DEGs. The protein‐protein interaction network was constructed by STRING 11.0 to reveal the potential gene interactions. The expression of cyclin‐dependent kinase 1 (Cdk1) at the messnger RNA (mRNA) and protein levels was examined by real‐time polymerase chain reaction (PCR) and Western blot analysis analysis, respectively.

**Results:**

The GEO database was searched, and the gene expression profiles of GSE55986 and GSE68539 were downloaded. A total of 262 DEGs and 356 DEGs were identified from GSE55986 and GSE68539, respectively. We found that the p53 signaling pathway might play a key role in the development of chemical cystitis, and Cdk1 acted as a crucial gene in the p53 signaling pathway. Moreover, the experimental results of real‐time PCR and Western blot analysis analysis demonstrated that the expression of Cdk1 at the mRNA and protein levels in cystitis tissues was significantly increased in different animal models of chemical cystitis compared with the control group.

**Conclusion:**

Cdk1 might be a potential pathogenic genetic target for chemical cystitis.

## Graphical abstract

Cdk1 shows to be a potential pathogenic genetic target for chemical cystitis

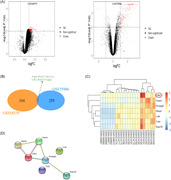

**Abbreviations**
Cdk1: cyclin‐dependent kinase 1
CYP: cyclophosphamide
DEGs: differential expression genes
GEO: Gene Expression Omnibus
IC: interstitial cystitis
LPS: lipopolysaccharide

## 1 BACKGROUND

Chemical cystitis becomes increasingly common with economic and social development. It is an important type of nonbacterial bladder inflammation. The most familiar clinical symptoms of chemical cystitis are unexplained urinary frequency and pelvic pain.^1^, ^2^, ^3^ Due to the unknown etiology and pathogenesis, treatment options lack pertinence, and the curative effect remains poor.^4^, ^5^ It is necessary to identify similar targets with different etiologies to better understand the molecular mechanisms of chemical cystitis. This also helps the clinical doctors apply some pertinent therapeutic measures to improve the life quality of patients.

As classic animal models of chemical‐induced cystitis, ketamine‐induced model, and cyclophosphamide (CYP)‐induced model can reflect the pathological progression, which are widely applied in the related basic research of nonbacterial bladder inflammation.^6^, ^7^ With the development of the gene detection technique, increasing attention has been paid to the changes in the related genes.^8^, ^9^ Gene Expression Omnibus (GEO) database is a commonly used functional gene data repository. In the present study, we downloaded the gene expression profiles of GSE55986 and GSE68539 from the GEO database. GSE55986 shows the gene expression data of the CYC‐induced mouse model of cystitis, while GSE68539 reveals gene expression data of the ketamine‐induced mouse model. GEO2R, the online tool of the GEO database, was used to identify the differentially expressed genes (DEGs) of GSE55986 and GSE68539. Moreover, the Kyoto Encyclopedia of Genes and Genomes (KEGG) pathway analysis and Gene Ontology (GO) function analysis of DEGs were performed by the Database for Annotation, Visualization, and Integrated Discovery (DAVID) version 6.8 Beta and R software. The analyzed outcomes revealed that the p53 signaling pathway was the key shared pathway between the ketamine‐induced model and CYC‐induced model. Besides, cyclin‐dependent kinase 1 (Cdk1) was found to be the only shared gene involved in the p53 signaling pathway of both models.

Some trials have shown that the mouse model induced by lipopolysaccharide (LPS) is reliable as it can also reflect the pathology of chemical‐induced cystitis.^10^, ^11^ To confirm whether Cdk1 was the common potential genetic target of chemical cystitis, Western blot analysis analysis, and polymerase chain reaction (PCR) were further performed in three models, including the ketamine‐induced model, CYC‐induced model, and LPS‐induced model. Following bioinformatic results, our experimental results revealed that the expression of Cdk1 was significantly increased in these three models. Therefore, we believed that Cdk1 functioned as a potential pathogenic genetic target for chemical cystitis.

## 2 METHODS

### 2.1 Microarray data

The series matrix files of GSE55986 and GSE68539 were downloaded from the GEO database. The platform of GSE55986 was GPL6246 (MoGene‐1_0‐st) Affymetrix Mouse Gene 1.0 ST Array (transcript [gene] version), while the platform of GSE68539 was GPL15523 Phalanx Mouse OneArray Ver 2. Three normal samples (GSM1349866, GSM1349867, and GSM1349868) and three CYP‐induced samples (GSM1349869, GSM1349870, and GSM1349871) were extracted from the GSE55986 profile. Eight normal samples (GSM1674776, GSM1674777, GSM1674778, GSM1674779, GSM1674784, GSM1674785, GSM1674786, and GSM1674787) and eight ketamine‐induced samples (GSM1674780, GSM1674781, GSM1674782, GSM1674783, GSM1674788, GSM1674789, GSM1674790, and GSM1674791) were extracted from the GSE68539 profile. R software was adopted to evaluate the data quality.

### 2.2 Identification of DEGs

DEGs were screened by GEO2R, which allowed users to compare two or more samples of the GEO series to identify genes expressed under different experimental conditions. The adjusted *p* values (adj. *p* value) and Log fold change (|logFC|) were important statistical indexes in the GEO2R results. If the adj. *p* value was less than .01 and |logFC| was greater than 1, the genes were identified as DEGs. Moreover, R ggplot2 and pheatmap were used to present these DEGs in the volcano plot and heatmap.

### 2.3 Functional enrichment analyses

The DAVID 6.8 Beta was used for functional enrichment analyses. First, the DEGs extracted from two GEO series were respectively up‐loaded to DAVID 6.8 Beta. Then the KEGG pathway analysis and GO enrichment analysis of DEGs were performed by the DAVID functional annotation tool. The results were expressed in bubble plots by using R software. *p *< .01 was considered statistically significant.

### 2.4 The construction of the protein–protein interaction (PPI) network

Search Tool for the Retrieval of Interacting Genes (version 11.0) is a database of known and predicted protein–protein interactions (PPIs), including direct (physical) and indirect (functional) associations. In our present study, the PPI network of seven co‐DEGs was constructed by STRING 11.0 to reveal the potential gene interactions. To visualize the interactions between the shared gene and other genes involved in the p53 signaling pathway of two animal models, the related genes in the PPI network were presented respectively.

### 2.5 Animal experiments

Female wild‐type C57BL/6 mice (20–25 g) were obtained from HFK Bio‐Technology Company (Beijing, China) and housed in the SPF‐grade animal facility under the controlled conditions as follows: 70% ± 4% relative humidity, 24 ± 1°C and a 12‐h light/dark cycle. The mice were given free access to tap water and food. All the animal‐related procedures were conducted following the guidelines established by the Institutional Animal Care and Use Committee of Suzhou Ninth Hospital Affiliated to Soochow University (wj2016065), and all animal‐related experiments were performed following the ARRIVE guidelines. In the ketamine‐induced model, the mice were administered with 30 mg/kg/day ketamine (Sigma‐Aldrich) via intraperitoneal injection for 12 weeks as previously described.^12^ The mice in its control group were injected with normal saline. In the CYP‐induced model, the mice were administered with 300 mg/kg/day CYP (Thermo Company) via intraperitoneal injection for 7 days as previously described,^13^ and the mice in its control group were injected with normal saline. In the LPS‐induced model, the mice were administered with 25 mg/kg LPS (Thermo Company) via intraperitoneal injection for 24 h as previously described. The mice in its control group were injected with normal saline. The mice were euthanized by carbon dioxide inhalation, and the bladders were removed after treatment.^10^

### 2.6 Quantitative PCR (qPCR)

Cystitis tissues were harvested after animal experiments, immediately placed on dry ice, and stored at −80°C (*n* = 3–5 in each group). Quantitative PCR (qPCR) was performed according to the instructions. Trizol Reagent was obtained from Invitrogen and used to isolate total RNA. Subsequently, 1 µg purified RNA was reversely transcribed into complementary DNA using Revert Aid First Strand cDNA Synthesis Kit (Fermentas). qPCR was carried out on an ABI 7500 system (Applied Biosystems) by using SYBR green PCR Master Mix (Invitrogen). The relative expression of Cdk1 was calculated using the 2‐∆∆Ct method, and glyceraldehyde 3‐phosphate dehydrogenase was selected as a housekeeping gene.

### 2.7 Western blot analysis analysis

Mouse cystitis tissue was homogenized in a lysis buffer supplemented with 1% protease inhibitor cocktail. The protein concentration was determined by a BCA protein assay kit (Pierce). Equal amounts of proteins were subjected to sodium dodecyl sulfate‐polyacrylamide gels using 10% gels and electrotransferred onto nitrocellulose membranes. The blots were blocked in 5% (wt/vol) nonfat dry milk in 0.1% Tris‐buffered saline/Tween 20 (TBST) for 1 h, followed by incubation with primary antibodies against CDK1 (Invitrogen) at a dilution 0.5 µg/ml and β‐actin (3700S; Cell Signaling Technology) at a dilution of 1:1000 overnight at 4°C. The membranes were washed with TBST and incubated with secondary antibodies (TIANGEN) for 1 h. β‐actin was used as a loading control. Immunoreactive bands were visualized using ECL chemiluminescence (Thermo Company), and the band densities were analyzed using ImageJ software (National Institute of Health).

### 2.8 Statistical analysis

All data were presented by mean ± *SEM*. Statistical analysis was performed by SPSS 23.0 Statistics software using Student's *t* test. *p* < .05 was considered statistically significant.

## 3 RESULTS

### 3.1 Data quality assessment and identification of DEGs

**Figure 1:**
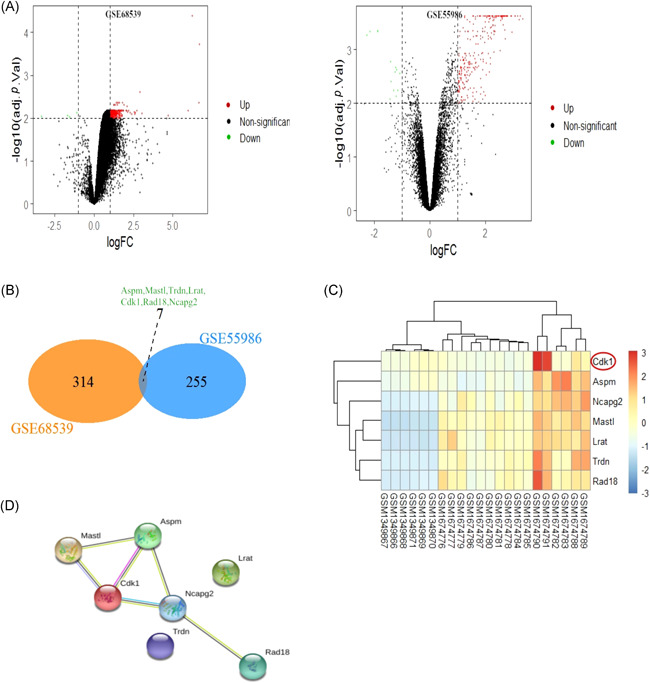
Screening of differential expression genes (DEGs) and protein–protein interaction (PPI) network of seven co‐DEGs. GSE55986 represents the cyclophosphamide (CYP)‐induced model. GSE68539 represents the ketamine‐induced model. (A) Volcano plot showing DEGs of CYP‐induced model and DEGs of ketamine‐induced model, respectively. (B) Venn diagram presents seven co‐genes of CYP‐induced model DEGs and ketamine‐induced model DEGs. (C) Heatmap reveals the expression changes of seven co‐DEGs. (D) PPI network reveals the gene interaction of seven co‐DEGs by STRING databaset's

A total of 262 DEGs and 356 DEGs were identified from GSE55986 and GSE68539 using the GEO2R online tool, respectively. CYP‐induced DEGs and ketamine‐induced DEGs were respectively visualized in the volcano plot (Figure 1A). Venn analysis was applied to the DEGs of two datasets, and there were seven co‐DEGs. Venn analysis results were presented in the Venn diagram (Figure 1B). The expressions of seven co‐DEGs were visually shown in the heatmap (Figure 1C). STRING database was used to construct the PPI network of seven co‐DEGs to reveal the potential gene interactions (Figure 1D). The PPI network showed that the hub genes might be Cdk1, Mastl, Aspm, and Ncapg2.

### 3.2 GO functional and KEGG pathway enrichment analyses

**Figure 2:**
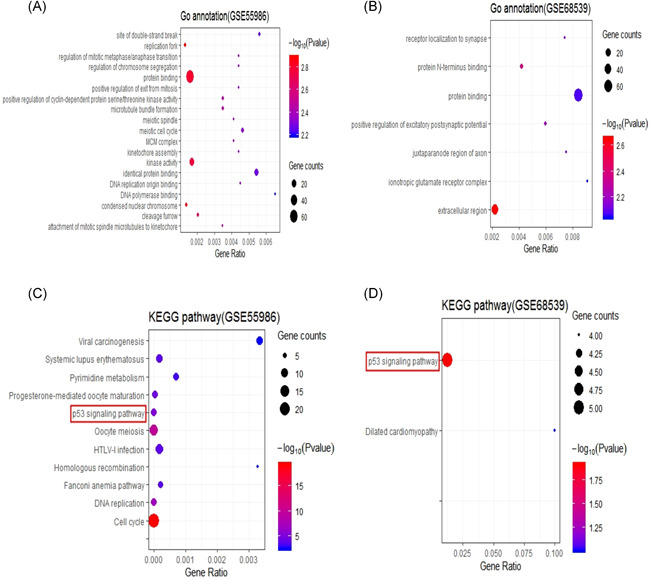
GO functional and KEGG pathway enrichment analyses of DEGs of CYP‐induced model and ketamine‐induced model. GSE55986 represents the CYP‐induced model. GSE68539 represents the ketamine‐induced model. (A, B) Bubble plot reveals the GO functional enrichment of DEGs of CYP‐induced model and ketamine‐induced model, respectively. (C, D) Bubble plot reveals the KEGG pathways of DEGs of CYP‐induced model and ketamine‐induced model, respectively. CYP, cyclophosphamide; DEG, differential expression gene; GO, Gene Ontology; KEGG, Kyoto Encyclopedia of Genes and Genomes

GO functional enrichment analysis of the DAVID database has three aspects, namely biological processes (BPs), molecular functions (MFs), and cellular components (CCs). According to the results of functional enrichment analyses, the GO terms were significantly representative if the *p* value was ≤.01. In our present study, 262 DEGs extracted from GSE55986 were significantly enriched in 64 BPs, while 356 DEGs extracted from GSE68539 were significantly enriched in two BPs. The most enriched GO terms of 262 DEGs were respectively related to the positive regulation of cyclin‐dependent protein serine/threonine kinase activity and microtubule bundle formation in BP, protein binding and kinase activity in MF, and replication fork and condensed nuclear chromosome in CC (Figure 2A). The most enriched Go terms of 356 DEGs were respectively related to the positive regulation of excitatory postsynaptic potential protein in BP, N‐terminus binding in MF, and extracellular region in CC (Figure 2B). Besides, the results of the KEGG pathway enrichment analysis were also shown. The KEGG terms were significantly representative if the *p* value was ≤.01. The 262 DEGs extracted from GSE55986 were significantly enriched in 11 KEGG pathway terms (Figure 2C), while 356 DEGs extracted from GSE68539 were significantly enriched in two KEGG pathway terms (Figure 2D).

### 3.3 p53 signaling pathway may be a key pathway in the development of chemical cystitis

KEGG pathway analysis was performed in DAVID using the bioinformatic approach. A total of 18 KEGG pathways were detected in both GEO series. There was only one shared KEGG pathway between the two series, namely the p53 signaling pathway. Venn diagram showed the only KEGG pathway by Venn analysis.

### 3.4 Cdk1 acts as a crucial gene in the p53 signaling pathway in chemical cystitis

**Figure 3:**
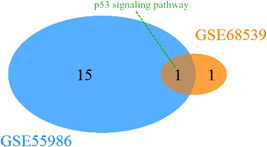
Venn diagram reveals the only shared KEGG pathway of CYP‐induced model DEGs and ketamine‐induced model DEGs. GSE55986 represents the CYP‐induced model. GSE68539 represents the ketamine‐induced model. CYP, cyclophosphamide; DEG, differential expression gene; KEGG, Kyoto Encyclopedia of Genes and Genomes

**Figure 4:**
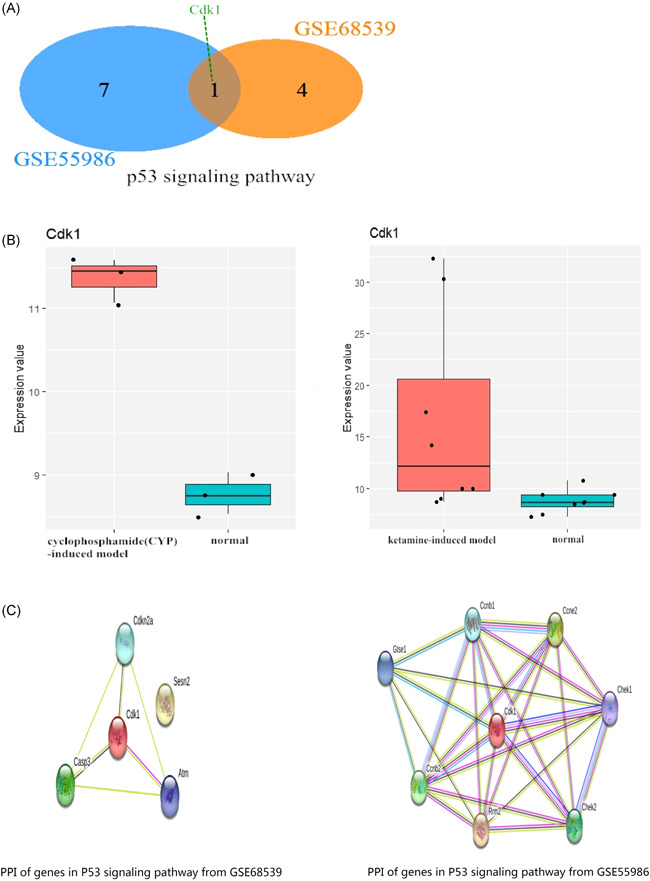
(A) Venn diagram reveals that cyclin‐dependent kinase 1 (Cdk1) is the co‐gene in the p53 signaling pathway. GSE55986 represents the CYP‐induced model. GSE68539 represents the ketamine‐induced model. (B) Box plot shows the expression change of Cdk1 in CYP‐induced model and ketamine‐induced model, respectively. (C) PPI network reveals the potential gene interaction in the p53 signaling pathway from two datasets, respectively. CYP, cyclophosphamide; PPI, protein–protein interaction

Venn analysis was performed to analyze the related genes in the p53 signaling pathway of the two models (Figure 3). The analytical results revealed that Cdk1 was the co‐gene in the p53 signaling pathway (Figure 4A). Subsequently, we found that the expression of Cdk1 was changed in the ketamine‐induced model and CYC‐induced model. Moreover, the expression changes of the two datasets were respectively shown in the Box plot (Figure 4B). This finding indicated that cdk1 might act as a potential genetic target. Next, the PPI network of the related genes involved in the p53 signaling pathway was constructed by using the STRING tool (Figure 4C), showing the network relationship between Cdk1 and other related genes in this pathway.

### 3.5 Upregulation of Cdk1 in the animal models of chemical cystitis

**Figure 5:**
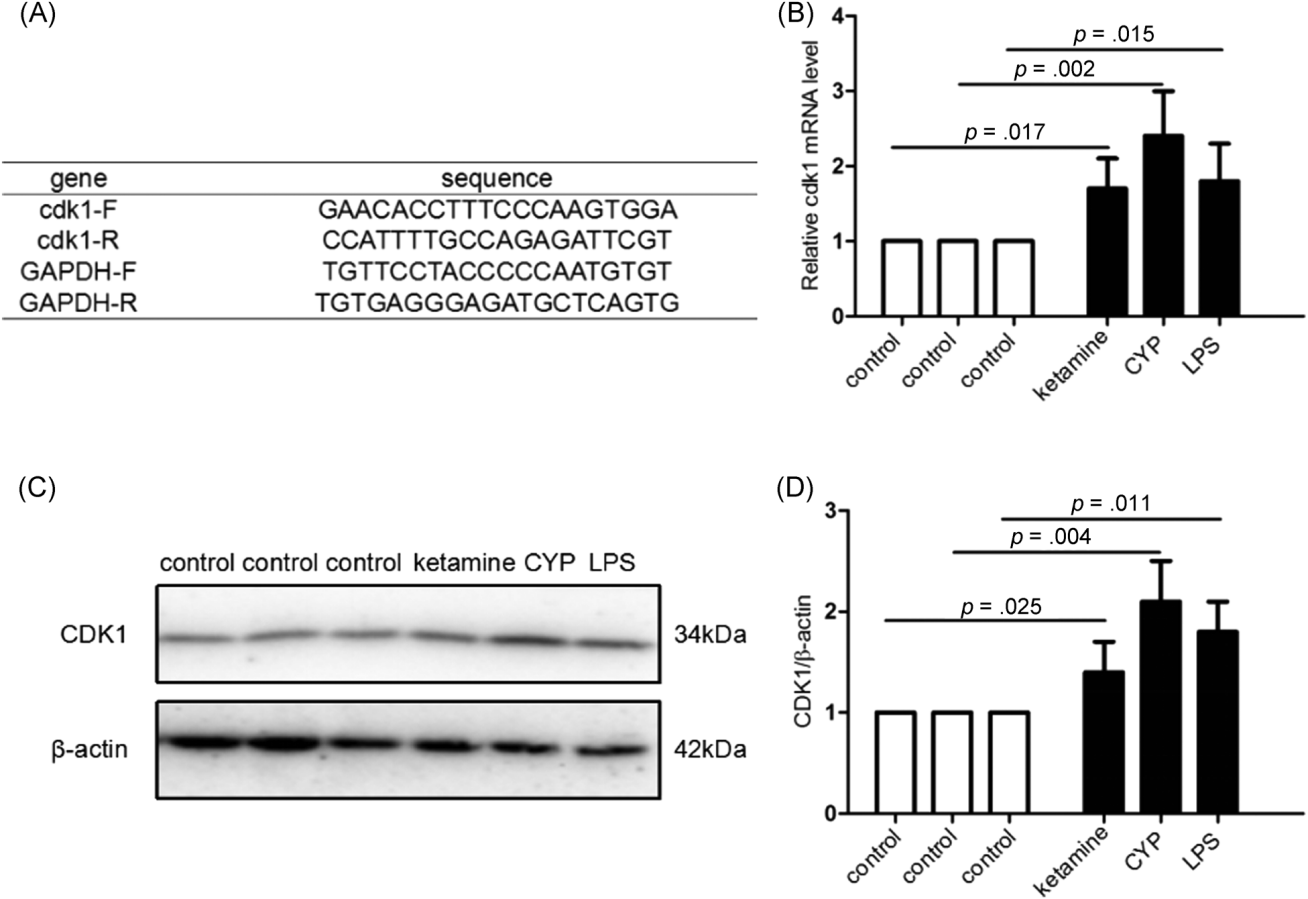
The expression of Cdk1 was verified by qPCR and Western blot analysis analysis. (A) The primer sequences of Cdk1 and GAPDH. (B) The expression of Cdk1 at the mRNA level was examined by qPCR analysis in three models. (C, D) The expression of Cdk1 at the protein level was detected by Western blot analysis analysis in three models. The statistical analysis of the expression of Cdk1 at the protein level. GAPDH was used as a loading control. Results were presented as mean ± *SEM*, and *p* < .05 was considered statistically significant. *n* = 5 mice for each treated group and *n* = 3 mice for each control group. Cdk1, cyclin‐dependent kinase 1; CYP, cyclophosphamide; GAPDH, glyceraldehyde 3‐phosphate dehydrogenase; LPS, lipopolysaccharide; mRNA, messenger RNA; qPCR, quantitative polymerase chain reaction

We studied the effects of Cdk1 expression in different animal models. Gene expression of Cdk1 was validated by qPCR and Western blot analysis analysis. Figure 5A‐D demonstrates that the expression of Cdk1 at the mRNA and protein levels in the cystitis tissue was significantly increased in animal models compared with the control group.

## 4 DISCUSSION

The unknown pathogenesis of chemical cystitis remains a challenge for clinicians, and a better understanding of the molecular mechanism of this disease is essential for developing more effective treatments.^14^ In our current study, we analyzed the genetic data of two classic cystitis animal models by using bioinformatic methods. We found that the p53 signaling pathway was shared in the pathological process by the two models. Cdk1 was the only shared gene in this pathway. Moreover, we further verified that Cdk1 was a key gene for the pathogenesis of chemical cystitis through the related experimental methods.

Our bioinformatic study showed that the p53 signaling pathway might be the key molecular pathological mechanism no matter in the ketamine‐induced model or CYC‐induced model. Our data indicated that the p53 signaling pathway played a key role in the disease progression of chemical‐induced cystitis. It is well known that p53 is a type of tumor suppressor protein.^15^ Many physiological and pathological activities of p53 are related to its tumor‐suppressive function, such as angiogenesis, promotion of senescence, apoptosis, and autophagy, and reactive oxygen species‐mediated DNA‐damage repair.^16^ However, the non‐cancer functions of the p53 signaling pathway have also been studied in‐depth, especially its mediating role in the expressions of specific genes for tight junction and adherens junction proteins.^17^ Some studies have revealed that compared with epithelial cells of the normal bladder, the expression of p53 is significantly increased in bladder epithelial cells from patients with interstitial cystitis (IC). As its active form, the level of phospho‐p53 is also significantly increased.^18^, ^19^, ^20^ It can regulate the expression of ZO‐1 involved in tight junction formation and E‐cadherin involved in cell adhesion by binding to their gene enhancers. Furthermore, p53 can significantly suppress the expression of ZO‐1 and increase the expression of E‐cadherin, leading to the non‐oncogenic pathophysiological changes of IC epithelial cells,^8^ such as increased paracellular permeability. The above‐mentioned experimental data further verify our bioinformatic study, suggesting that the p53 signaling pathway plays a key role in the development of chemical‐induced cystitis. Besides, we explored the core gene that could phosphorylate p53 in this pathway by further research.

According to our study, Cdk1 was the core gene in the p53 signaling pathway, and some previous studies have revealed that Cdk1 can phosphorylate p53.^21^, ^22^ This finding indicated that Cdk1 might play a key role in the disease development of chemical‐induced cystitis. To confirm such a finding, the related experiments were adopted in our study. Our experimental results were consistent with the bioinformatic findings. In addition to the ketamine‐induced model and CYC‐induced model, the IC model induced by LPS was also included in our experiment. The research on these three classical animal models would increase the credibility of our experimental results. Our experiment revealed that the expression of Cdk1 at the RNA and protein levels was significantly increased in all three models. About the specific mechanism of Cdk1 in the p53 signaling pathway, some previous studies have shown that Cdk1 can phosphorylate p53 in irradiated human colon cancer HCT116 cells. Cdk1 can be combined with cyclin B1, forming the cyclin B1/Cdk1 complex.^23^ Then the complex is translocated into mitochondria. The mitochondrial cyclin B1/Cdk1 is found to phosphorylate p53 at Ser‐315 residues.^24^ Certainly, the specific mechanism by which Cdk1 phosphorylates p53 in chemical‐induced cystitis needs to be verified in further studies.

## 5 CONCLUSION

Collectively, our bioinformatic analysis of two animal models revealed that the p53 signaling pathway was a key biological pathway in the disease progression of chemical‐induced cystitis. Moreover, Cdk1 was identified as a potential pathogenic genetic target for chemical cystitis according to bioinformatic analysis and experimental study. Taken together, our findings provided valuable insights into the molecular mechanisms of chemical cystitis.

## CONFLICT OF INTERESTS

The authors declare that there are no conflict of interests.

## AUTHOR CONTRIBUTIONS

Kun Wang and Huaping Du conceived and designed the study. Dong Xue and Kun Wang performed the experiment and prepared Figures 1, 2, 3, 4, 5. Kun Wang and Qianfeng Zhuang wrote the main manuscript text. Huaping Du, Renfang Xu, and Hao Lu reviewed and edited the manuscript. All authors read and approved the manuscript.

## ETHICS STATEMENT

All the procedures of animal experiments were in accordance with the guidelines established by the Institutional Animal Care and Use Committee of Soochow University.
